# Chemogenetic Activation of LC Noradrenergic Afferents Facilitates Cerebellar CF–PC LTD via Presynaptic α_2_A–AR/CDK5/PKA Signaling

**DOI:** 10.3390/biom16071042

**Published:** 2026-07-17

**Authors:** Xu-Dong Zhang, Ying-Han Xu, Wang-Tong Wu, Lang-Yue Zheng, Xin-Yi Xu, Chun-Ping Chu, De-Lai Qiu

**Affiliations:** 1 Department of Physiology and Pathophysiology, College of Medicine, Yanbian University, Yanji 133002, China; 2022001069@ybu.edu.cn (X.-D.Z.); 2022001068@ybu.edu.cn (Y.-H.X.); 2 Institute of Brain Science, Jilin Medical University, Jilin City 132013, China; wwt17778598923@gmail.com (W.-T.W.); meugmok@gmail.com (L.-Y.Z.); xxinyi323@gmail.com (X.-Y.X.); 3 Department of Physiology, College of Basic Medicine, Jilin Medical University, Jilin City 132013, China

**Keywords:** cerebellum, locus coeruleus, chemogenetic activation, α_2_-adrenergic receptor, climbing fiber–Purkinje cell synaptic plasticity, cyclin–dependent kinase 5 (CDK5), protein kinase A (PKA)

## Abstract

Cerebellar climbing fiber–Purkinje cell (CF–PC) long-term depression (LTD) plays a critical role in motor learning and is modulated by locus coeruleus (LC) noradrenergic afferents via distinct adrenergic receptor (AR) subtypes. Nevertheless, the mechanisms underlying LC noradrenergic neuron-mediated regulation of CF–PC LTD remain poorly understood. Here, we investigated the effects of chemogenetic activation of LC noradrenergic afferents on CF–PC LTD in cerebellar slices from dopamine β-hydroxylase (DBH)-Cre mice using electrophysiology, glutamate sensor imaging, immunofluorescence and pharmacological approaches. Tetanic stimulation (5 Hz) of CFs induced CF–PC LTD under control conditions, and this LTD was enhanced by chemogenetic activation of LC noradrenergic afferents. Blockade of group I metabotropic glutamate receptors (mGluR1) abolished LTD under control conditions, whereas chemogenetic activation of LC noradrenergic afferents triggered a novel form of CF–PC LTD accompanied by an increased N2/N1 ratio. With mGluR1 blocked, chemogenetic activation of LC noradrenergic afferents failed to trigger the novel CF–PC LTD following blockade of α_2_-AR or α_2_A-AR, but not α_2_B-AR or α_2_C-AR. Importantly, chemogenetic activation of LC noradrenergic afferents triggered LTD of glutamate fluorescence at CF terminals, which was abolished by blockade of α_2-_AR or α_2_A-AR, but not α_2_B-AR or α_2_C-AR. Notably, inhibition of either cyclin-dependent kinase 5 (CDK5) or presynaptic, but not postsynaptic, protein kinase A (PKA) completely abolished the CF–PC LTD triggered by chemogenetic activation of LC noradrenergic afferents in mouse cerebellar slices. Immunofluorescence results showed robust α_2_A-AR expression throughout the cerebellar molecular layer, with intense signals along PC dendrites and clear colocalization with vesicular glutamate transporter 2 (vGluT2) at cerebellar CF terminals. These results indicate that activation of LC noradrenergic afferents potentiates CF–PC LTD by triggering Glu-LTD at CF terminals through the α_2_A-AR/CDK5/PKA signaling cascade in the mouse cerebellar cortex.

## 1. Introduction

In the cerebellar cortex, climbing fibers (CFs) arise from the inferior olive (IO) of the medulla oblongata and constitute one of the two major excitatory glutamatergic inputs to Purkinje cells (PCs) [1]. CF terminals make hundreds of synaptic contacts along the proximal dendrites of PCs, and a single CF action potential evokes large-amplitude excitatory postsynaptic currents (EPSCs) and extensive dendritic Ca^2+^ transients [2,3,4]. Accordingly, the CF–PC synapse is widely regarded as a critical synaptic substrate within the cerebellar cortex for relaying error signals, modulating the spontaneous firing and output of PCs, and involving motor learning [4,5,6]. CF–PC long-term depression (LTD) was first reported by Hansel and Linden. Their work showed that brief 5 Hz stimulation of CFs in cerebellar slices induces a sustained reduction in the amplitude of evoked EPSCs. This form of CF–PC LTD relies on postsynaptic Ca^2+^ influx, activation of group I metabotropic glutamate receptors (mGluR1), and protein kinase C (PKC) signaling [7]. In addition, CF–PC LTD reduces CF-mediated synaptic currents, and weakens the slow phase of complex spikes as well as CF-evoked Ca^2+^ transients within PC dendrites [8]. Our prior work demonstrated that tetanic stimulation of CFs induces mGluR1-dependent CF–PC LTD in mouse cerebellar slices, a process regulated by the (cannabinoid receptor type 1) CB1 receptor/protein kinase A (PKA) signaling cascade [9]. CF–PC LTD allows PCs to adapt to physiological excitatory inputs from CFs at the synaptic level. It also supports the instructive function of CF-derived error signals in cerebellum-dependent motor learning, as well as neural processing associated with motor adaptation and memory [7,8,10,11,12].

Anatomical studies have shown that noradrenergic neurons of the pontine locus coeruleus (LC) give rise to extensive projections to the cerebellar cortex and cerebellar nuclei. Therefore, the LC noradrenergic system can exert broad influences on neuronal excitability, synaptic transmission and synaptic plasticity in the cerebellum via different adrenergic receptors (ARs) [13,14,15,16,17]. ARs are G protein-coupled receptors divided into three main families: α_1_, α_2_ and β receptors. Each family contains multiple subtypes, namely α_1_A, α_1_B, α_1_D, α_2_A–α_2_C, and β_1_–β_3_ [18]. All AR subtypes have been detected in the cerebellum, where distinct AR subtypes differentially regulate neuronal activity throughout the cerebellar cortex [13]. In situ hybridization further revealed that, among α_2_-AR subtypes, transcripts of α_2_A-AR and α_2_B-AR are widely distributed across neuronal populations in the cerebellar cortex [19].

Noradrenaline (NA) suppresses CF-evoked dendritic Ca^2+^ signals in PCs via α_2_-ARs. Activation of α_2_-ARs reduces glutamate release from CF axonal terminals, attenuates CF-evoked calcium transients in PC dendrites, and diminishes endocannabinoid release, thereby impairing short- and long-term plasticity at PF–PC synapses [20]. Our previous studies demonstrated that NA suppresses spontaneous complex spike activity in PCs and inhibits CF–PC synaptic transmission via α_2_-ARs. This process relies on the presynaptic adenylyl cyclase (AC)–PKA signaling cascade, indicating that α_2_-ARs modulate synaptic transmission in the CF–PC pathway by lowering glutamate release probability at CF terminals [21,22]. Furthermore, NA elevates basal endogenous γ-aminobutyric acid (GABA) release and decreases glutamate release in the rat hippocampus in a concentration-dependent manner upon α_2_-AR activation [23]. Moreover, α_2_A-ARs are abundantly expressed in the granular layer of the mouse cerebellum. In vivo studies in mice revealed that these receptors mediate the suppression of mossy fiber–granule cell (MF–GrC) long-term potentiation (LTP) triggered by optogenetic activation of LC noradrenergic neurons, through the induction of presynaptic LTD [24]. Collectively, ARs are essential for synaptic transmission and plasticity at CF–PC synapses within cerebellar neuronal circuits, suggesting that noradrenergic fibers originating from the LC modulate CF–PC synaptic function via distinct AR subtypes. However, the mechanisms underlying LC noradrenergic regulation of CF–PC LTD remain unclear. We therefore investigated how LC noradrenergic afferents regulate long-term synaptic plasticity at CF–PC synapses in cerebellar slices from dopamine β-hydroxylase (DBH)-Cre mice using electrophysiology, glutamate sensor imaging, immunofluorescence and pharmacological approaches.

## 2. Materials and Methods

### 2.1. Animals Preparation

#### 2.1.1. Animals

A total of 84 DBH-Cre mice (2–3 weeks old, equal males and females, stock #T005671) were obtained from GemPharmatech Co., Ltd. (Nanjing, China) and used for projection mapping and in vitro slice experiments. All animals were group-housed under a 12 h light–dark cycle (lights on: 06:00–18:00), with ad libitum access to food and water. The housing temperature was maintained at 24 ± 1 °C and humidity at 50 ± 5%. For electrophysiological recordings, these mice (even sex distribution) were randomly assigned to control and drug-treated groups. All animal experiments were reviewed and approved by the Animal Care and Use Committee of Yanbian University (Permit No.: SYXK (Ji) 2025–0005, issued on 11 July 2025). Procedures were carried out following the National Institutes of Health Guidelines for the Care and Use of Laboratory Animals, as well as the Animal Research: Reporting of In Vivo Experiments guidelines (ARRIVE; https://arriveguidelines.org (accessed on 14 July 2020)).

#### 2.1.2. Stereotaxic Surgery, Viral Nanoinjection and Chemogenetic Activation of Noradrenergic Afferents

**Figure 1:**
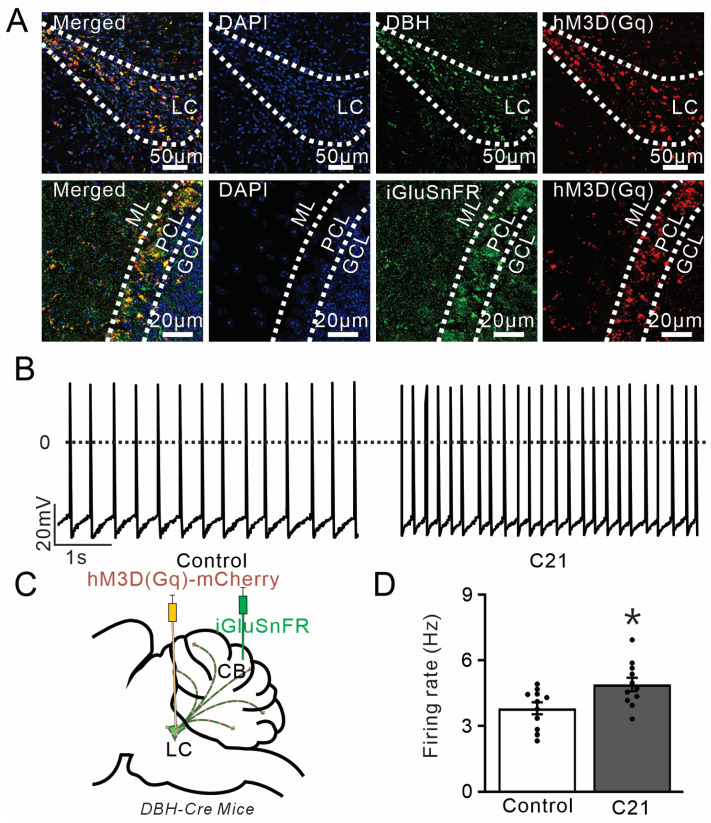
Viral expression of hM3D(Gq) in LC noradrenergic neurons and iGluSnFR in the cerebellum of DBH-Cre mice. (**A**) Representative immunofluorescence images display hM3D(Gq)–mCherry expression in the LC and iGluSnFR expression in the cerebellum. (**Upper panels**) present hM3D(Gq)–mCherry-positive LC neurons co-labeled with DBH and DAPI. (**Lower panels**) illustrate cerebellar iGluSnFR signals alongside hM3D(Gq)–mCherry and DAPI staining. Red: hM3D(Gq)–mCherry; green: DBH or iGluSnFR; blue: DAPI. (**B**) Representative traces depict spontaneous firing activity of LC noradrenergic neurons at baseline and during C21-evoked chemogenetic activation. (**C**) Schematic diagram illustrating viral delivery of hM3D(Gq) and iGluSnFR into the LC of DBH-Cre mice. The colors represent the fluorescence tags: mCherry (yellow) and iGluSnFR (green). (**D**) Mean value (±S.E.M) and individual data points show the spontaneous firing rates of LC noradrenergic neurons under control conditions and C21 treatment. * *p* < 0.05 versus control. Control group: *n* = 11 recordings from 11 slices of 6 mice; C21 group: *n* = 11 recordings from 11 slices of 6 mice.

Stereotaxic surgery and nanoinjection were performed as previously described [24]. Briefly, DBH-Cre mice were anesthetized in an induction chamber using 4% isoflurane (R500, RWD, Shenzhen, China) and secured in a stereotaxic frame under sterile conditions (68801, RWD, Shenzhen, China). Anesthesia was maintained with 1–2% isoflurane throughout the entire surgical procedure. For in vitro electrophysiological experiments, DBH-Cre mice received unilateral injections of 300 nL glutamate sensor virus (AAV2/9–hSyn–SF–iGluSnFR.A184S–WPRE, Obio Technology, Shanghai, China) at two sites in the cerebellar vermis [AP: −7.0 mm, ML: 0.0 mm, DV: 1.0 mm and AP: −8.0 mm, ML: 0.0 mm, DV: −1.5 mm]. In experiments involving chemogenetic activation of LC noradrenergic neurons, mice were additionally injected bilaterally with 300 nL of AAV2/9–hSyn–DIO–hM3D(Gq)–mCherry–WPRE–pA and AAV2/9–hSyn–DIO–mCherry–WPRE–pA (Taitool, Shanghai, China) per side into the LC (AP: −5.45 mm; ML: ±1.25 mm; DV: −3.8 mm). For anatomical tracing of CF projections from the IO to the cerebellar vermis, DBH mice received unilateral stereotaxic injections of 300 nL AAV2/1–vesicular glutamate transporter 2 (vGluT2)–EGFP–WPRE–hGH–pA (BrainVTA, Wuhan, China) into the IO (AP: −7 mm; ML: ±0.24 mm; DV: −5.6 mm). Viral injections were performed using a Nanoject III microinjector (Drummond Scientific, Broomall, PA, USA) equipped with glass micropipettes (tip diameter ~20 μm) fabricated by a micropipette puller (P97, Sutter Instrument, Novato, CA, USA). Viruses were infused at a rate of 1 nL/s. The pipette was kept in place for an additional 10 min after injection to minimize backflow. Following viral delivery, mice were given a recovery period of at least 4 weeks to ensure robust viral expression before chemogenetic and projection mapping experiments. Upon completion of in vivo electrophysiological recordings, mice were perfused for verification of viral expression and neural projections (Figure 1A).

To achieve chemogenetic activation of LC noradrenergic afferents in the cerebellar cortex, we bath-applied 1 μM C21, an agonist for Designer Receptors Exclusively Activated by Designer Drugs (DREADDs), to activate hM3D(Gq) [25]. C21 was dissolved in artificial cerebrospinal fluid (ACSF) containing 125 mM NaCl, 3 mM KCl, 1 mM MgSO_4_, 2 mM CaCl_2_, 1 mM NaH_2_PO_4_, 25 mM NaHCO_3_ and 10 mM D-glucose. The solution was continuously aerated with 95% O_2_/5% CO_2_ and bath-applied to cerebellar slices at a perfusion rate of 0.5 mL/min. To examine the effects of chemogenetic activation of LC noradrenergic afferents on CF–PC LTD, 5 Hz tetanic stimulation of CFs was delivered to mouse cerebellar slices in the presence of C21.

### 2.2. Cerebellar Slice Preparation and Whole-Cell Recording

Mice (*n* = 68) were anesthetized with isoflurane and promptly decapitated. Brains were rapidly dissected and transferred to ice-cold sucrose-based ACSF aerated with 95% O_2_ and 5% CO_2_. The sucrose-based ACSF consisted of (in mM): 200 sucrose, 3 KCl, 2 CaCl_2_, 2 MgCl_2_, 26 NaHCO_3_, 1.25 NaH_2_PO_4_ and 10 D-glucose (pH 7.4; 295–305 mOsm). Coronal pontine slices containing the LC and sagittal cerebellar slices (300 μm thick) were prepared using a vibrating microtome (Leica VT1200S; Leica Biosystems Nussloch GmbH, Nussloch, Germany). Slices were subsequently transferred to standard ACSF (with sucrose substituted by 124 mM NaCl) and allowed to recover for 1 h at room temperature (24–25 °C) prior to electrophysiological recording.

Whole-cell patch-clamp recordings were performed under visual guidance using an upright Nikon Eclipse FN1 microscope equipped with a 40× water-immersion objective. Recording pipettes were pulled from borosilicate glass capillaries (World Precision Instruments, Sarasota, FL, USA) using a PC-10 micropipette puller (Narishige, Tokyo, Japan). When filled with the internal solution, pipette resistance was between 3 and 5 MΩ. For whole-cell recordings of LC neurons, the intracellular solution contained (in mM): 130 potassium gluconate, 10 KCl, 10 HEPES, 2 Mg–ATP, 0.3 Na–GTP and 0.4 EGTA (pH 7.3; osmolarity 300 mOsm). For whole-cell patch-clamp recordings from the somata of cerebellar PCs, the intracellular solution consisted of (in mM): 128 CsOH, 111 gluconic acid, 10 CsCl, 0.5 EGTA, 4 NaOH, 2 MgCl_2_, 4 Na_2_ATP, 0.4 Na_2_GTP and 30 sucrose (pH adjusted to 7.25 with CsOH). All PCs were voltage-clamped at −70 mV. Series resistance was continuously monitored throughout recordings using a 5 mV, 10 ms voltage step. Only cells with stable series resistance were included in the analysis. Membrane currents and potentials were recorded with an Axopatch 700B amplifier (Molecular Devices, Foster City, CA, USA), low-pass filtered at 2 kHz, digitized via a Digidata 1440 A/D converter (Molecular Devices, Foster City, CA, USA), and acquired using Clampex 10.4 software (Molecular Devices, Foster City, CA, USA).

### 2.3. Glutamate Fluorescence Imaging

Four weeks after viral injection, acute cerebellar slices were prepared from DBH-Cre mice expressing the iGluSnFR.A184S glutamate sensor. Whole-cell patch-clamp recordings were acquired from PC somata in these slices, with simultaneous iGluSnFR fluorescence imaging. Slices were transferred to a recording chamber fixed on an upright Nikon Eclipse FN1 microscope and continuously perfused with oxygenated ACSF. Fluorescence signals were visualized via a 40× water-immersion objective (NIR Apo 40×/0.80 W DIC N2, WD 3.5 mm; Nikon, Tokyo, Japan). iGluSnFR.A184S was excited at 488 nm using a DG-4 wavelength–switching light source (Sutter Instrument, Novato, CA, USA). Fluorescence images were captured at 15 Hz by a Hamamatsu ORCA-Flash4.0 LT digital CMOS camera controlled by HCImage software (version 4.7.0; Hamamatsu Photonics, Hamamatsu, Japan). Each imaging acquisition lasted 3 s, with an intertrial interval of 30 s between consecutive trials.

Image stacks were analyzed offline using ImageJ software (version 2.14.0, National Institutes of Health, Bethesda, MD, USA). Background fluorescence was subtracted from each frame based on tissue-free regions with negligible fluorescent signals. Regions of interest (ROIs) were selected within the molecular layer (ML) covering the stimulation-evoked fluorescent response area. The baseline fluorescence (F_0_) was defined as the mean fluorescence intensity during the pre-stimulation window from −1 to 0 s. Fluorescence changes were calculated as ΔF/F = (F_t_ − F_0_)/F_0_ and presented as percentage values, where F_t_ indicates the fluorescence intensity at a given time point t and F_0_ corresponds to the mean fluorescence obtained during the 1 s baseline period prior to stimulation [26]. The peak ΔF/F amplitude and area under the curve (AUC) were quantified for subsequent statistical analysis.

### 2.4. Immunohistochemistry and Confocal Fluorescence Imaging

To examine the expression of AAV2/9–hSyn–SF–iGluSnFR.A184S–WPRE and AAV–DIO–hM3D(Gq)–mCherry and assess their colocalization with DBH, DBH-Cre mice were anesthetized via intraperitoneal injection of 2,2,2-tribromoethanol (250 mg/kg) four weeks after AAV delivery. The mice were then transcardially perfused with ice-cold phosphate-buffered saline (PBS), followed by 4% paraformaldehyde. Brains were dissected out and post-fixed overnight. Subsequently, tissues were dehydrated sequentially in 10%, 20% and 30% sucrose solutions at 4 °C. Coronal sections of the LC with a thickness of 40 μm were prepared using a CM1900 freezing microtome (Leica Microsystems GmbH, Wetzlar, Germany). The sections were first permeabilized with 0.3% Triton X-100 at room temperature for 15 min, and then blocked with 10% goat serum for 2 h. Afterwards, the sections were incubated overnight at 4 °C with rabbit anti-DBH primary antibody (Cat# A2711, 1:2000; ABclonal, Wuhan, China). Next, samples were incubated for 2 h at room temperature in the dark with donkey anti-rabbit DyLight 488 secondary antibody (Cat# SA5–10038, 1:500; Invitrogen, Carlsbad, CA, USA) and 4′,6-diamidino-2-phenylindole (DAPI; Cat# D3571, 1:1000, Thermo Fisher Scientific; Waltham, MA, USA). After three washes with PBS, the sections were mounted with anti-fade mounting medium. To visualize CF projections from the IO to the cerebellar cortex, mice received IO injections of AAV–vGluT2–EGFP (BrainVTA, Wuhan, China). Cerebellar vermis sections were immunostained with rabbit anti-α_2_A-AR antibody (Cat# A271, 1:1000; Sigma–Aldrich, St. Louis, MO, USA) followed by goat anti-rabbit Cyanine5 secondary antibody (Cat# A10523, 1:500; Invitrogen, Carlsbad, CA, USA). Fluorescence images were acquired using a Nikon A1RMP high-speed multiphoton confocal laser-scanning microscope (Tokyo, Japan).

### 2.5. Chemicals

C21 (hM3D(Gq) DREADD agonist) was purchased from MedChemExpress LLC (Monmouth Junction, NJ, USA). Picrotoxin, JNJ (selective mGluR1 antagonist), yohimbine hydrochloride (YHB; non-selective α_2_-adrenoceptor antagonist), BRL44408 (BRL; selective α_2_A-adrenoceptor antagonist), imiloxan hydrochloride (selective α_2_B-adrenoceptor antagonist), JP-1302 dihydrochloride (JP; selective α_2_C-adrenoceptor antagonist), roscovitine (cyclin-dependent kinase 5 (CDK5) inhibitor), KT5720 and protein kinase inhibitor-(6–22) amide (PKI) were bought from GLBIO (Montclair, NJ, USA). Picrotoxin (50 µM) was added to ACSF throughout all recordings to eliminate GABA_A_ receptor-mediated inhibitory currents. All drugs were bath-perfused onto cerebellar slices at a flow rate of 0.5 mL/min using a Gilson Minipulse 3 peristaltic pump (Villiers-le-Bel, France).

### 2.6. Statistical Analysis

All experimental data were first subjected to post hoc power analysis using G*Power 3.1. Sample sizes (*n*) for all comparisons were determined with a statistical power above 0.8 at a significance level of α = 0.05. Electrophysiological data were analyzed with Clampfit 11.6 (Molecular Devices, Foster City, CA, USA). The paired-pulse ratio (N2/N1; PPR) was calculated as the amplitude ratio of the second excitatory postsynaptic current (N2) to the first excitatory postsynaptic current (N1). The mean baseline values were calculated from data collected 10 min prior to 5 Hz tonic stimulation, while post-stimulation values were averaged from recordings obtained 30–40 min after stimulation offset. For normalization, the amplitudes and AUC of response waves, as well as iGluSnFR fluorescence signals before (Pre) and after (Post) 5 Hz tonic stimulation, were divided by the average baseline of each group and then multiplied by 100. All data are expressed as mean ± S.E.M. The normality of experimental data was examined using the Kolmogorov–Smirnov test, and parametric or non-parametric statistics were applied accordingly. Repeated-measures one-way ANOVA followed by Tukey’s post hoc test (SPSS 17.0, Chicago, IL, USA) was used for intergroup statistical comparisons. A *p*-value less than 0.05 was considered statistically significant.

## 3. Results

### 3.1. Chemogenetic Activation of LC Noradrenergic Afferents Enhances 5 Hz Stimulation-Induced Cerebellar CF–PC LTD

For selective activation of LC noradrenergic neurons, AAV–DIO–hM3D(Gq)–mCherry, a Cre-dependent viral vector, was stereotaxically injected into the LC of DBH-Cre mice (Figure 1C). hM3D(Gq), a Gq-coupled designer receptor derived from the human M3 muscarinic receptor, was thereby specifically expressed in DBH-positive noradrenergic neurons [24,27,28]. Fluorescence imaging confirmed robust hM3D(Gq)–mCherry expression within the LC, in line with the endogenous distribution of LC noradrenergic neurons (Figure 1A). Meanwhile, sparse punctate mCherry labeling was present in the ML of the cerebellar cortex, implying axonal innervation of the cerebellar cortex by LC noradrenergic neurons. Bath application of the DREADD agonist C21 (1 μM) markedly elevated the spontaneous firing rate of LC neurons from a baseline of 3.8 ± 0.3 Hz to 4.9 ± 0.3 Hz after drug treatment (F_(1,20)_ = 86.4, *p* < 0.001; *n* = 11 cells; Figure 1D). These findings confirm that functional hM3D(Gq) was expressed in LC noradrenergic neurons of DBH-Cre mice, and that their axonal projections extend to the ML of the cerebellar cortex.

**Figure 2:**
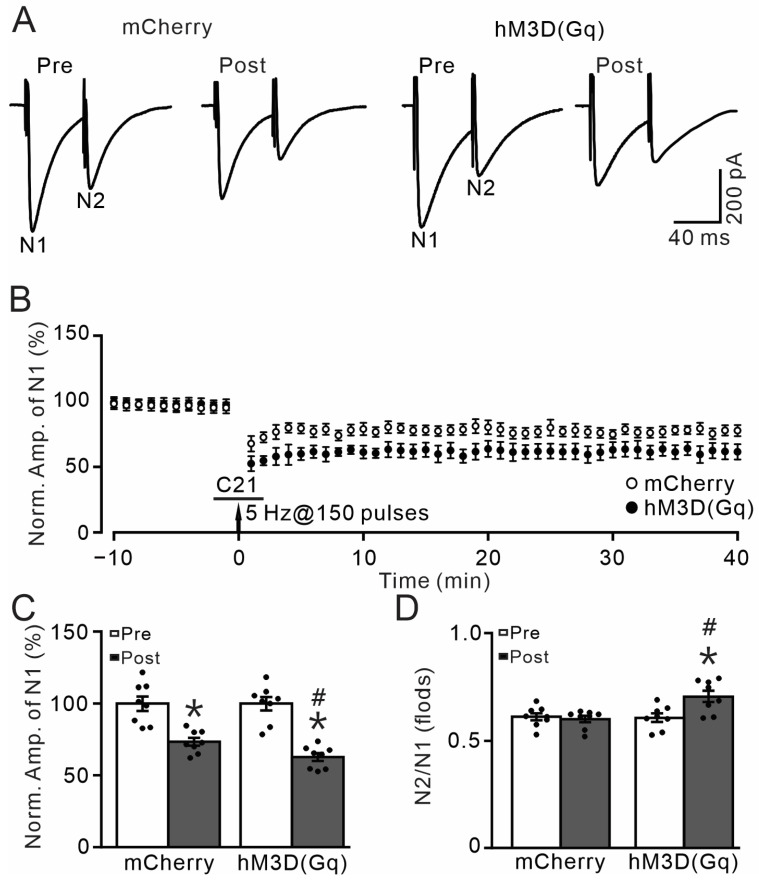
Chemogenetic activation of LC noradrenergic afferents enhances tetanus-evoked CF–PC LTD in mouse brain slices. (**A**) Representative traces show CF–PC EPSCs evoked by paired-pulse stimulation (0.05 Hz, 0.2 ms) of CF before (Pre) and after (Post) 5 Hz stimulation (150 pulses), under control conditions (mCherry) and chemogenetic activation of LC noradrenergic afferents with 1 μM C21 (hM3D(Gq)). (**B**) Summary time-course plots show normalized N1 amplitudes before and after 5 Hz CF stimulation (black arrow) in mCherry (open circles) and hM3D(Gq) (filled circles) groups. The horizontal bar indicates the period of C21 application. (**C**,**D**) Mean values (±S.E.M) and individual data points illustrate normalized N1 amplitude (**C**) and PPR (**D**) before (Pre) and after (Post) tetanic stimulation in each group. * *p* < 0.05 versus Pre; # *p* < 0.05 versus Post of control groups. Control group: *n* = 8 recordings/8 slices/6 mice; hM3D(Gq) group: *n* = 8 recordings/8 slices/6 mice.

Consistent with previous studies [7,9], tetanic stimulation (5 Hz, 150 pulses) induced LTD of CF–PC synaptic transmission in the presence of the GABA receptor antagonist picrotoxin (50 μM; Figure 2). Tetanic stimulation applied in the presence of the DREADD agonist C21 (1 μM) triggered a sustained reduction in the N1 amplitude of CF–PC EPSCs, which persisted for at least 40 min in the control group (mCherry; Figure 2A,B). During the 30–40 min period following tetanic stimulation, the normalized N1 amplitude decreased to 73.44 ± 2.78% of baseline (Pre: 100.0 ± 5.09%; F_(1,14)_ = 19.03, *p* < 0.001; *n* = 8 recordings; Figure 2C). The PPR was 0.60 ± 0.02, which was similar to baseline (Pre: 0.61 ± 0.02; F_(1,14)_ = 0.56, *p* = 0.48; *n* = 8 recordings; Figure 2D). These results show that 5 Hz stimulation combined with C21 reliably induces robust CF–PC LTD, with no significant change in PPR in cerebellar slices from control mice.

Notably, CF–PC LTD induced by tetanic stimulation combined with C21 was markedly enhanced in the cerebellar cortex of hM3D(Gq)-expressing mice (Figure 2A,B). During the 30–40 min period after tetanic stimulation, the normalized N1 amplitude in the hM3D(Gq) group decreased to 62.80 ± 2.76% of baseline (Pre: 100.0 ± 4.57%; F_(1,14)_ = 24.34, *p* < 0.001; *n* = 8 recordings; Figure 2C). This value was significantly lower than that in the control group (73.44 ± 2.78%; F_(1,14)_ = 7.37, *p* = 0.02; Figure 2C). However, the PPR was 0.71 ± 0.03 during the 30–40 min period after tetanic stimulation in the hM3D(Gq) group, which was significantly higher than the baseline (Pre: 0.61 ± 0.02; F_(1,14)_ = 72.9, *p* < 0.001; Figure 2D) and that in the control group (0.60 ± 0.02; F_(1,14)_ = 34.58, *p* < 0.001; Figure 2D). These results demonstrate that chemogenetic activation of LC noradrenergic afferents enhances the tetanic stimulation-induced CF–PC LTD, accompanied by an increase in the PPR in the mouse cerebellar cortex.

### 3.2. Chemogenetic Activation of LC Noradrenergic Afferents Triggers Tetanic Stimulation-Evoked CF–PC LTD via α_2_A-AR Signaling

**Figure 3:**
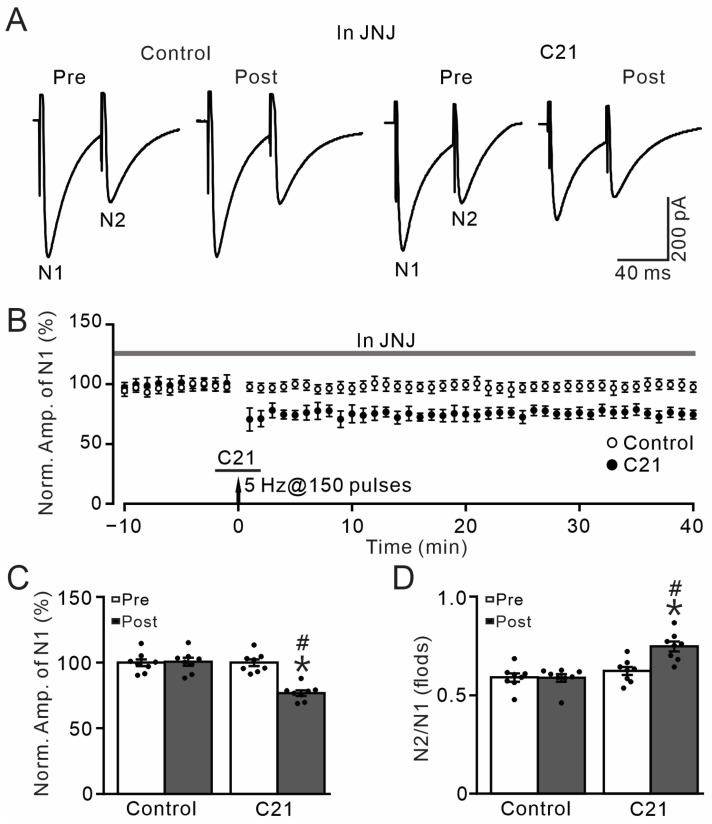
Blockade of mGluR1 abolished CF–PC LTD in the control group, yet unmasked a distinct form of LTD following chemogenetic activation of LC noradrenergic afferents. (**A**) In the presence of JNJ (1 μM), representative traces show CF–PC EPSCs evoked by paired-pulse stimulation of CF (0.05 Hz, 0.2 ms) before (Pre) and after (Post) 5 Hz tetanic stimulation (150 pulses) under control conditions and C21–induced chemogenetic activation of LC noradrenergic afferents. (**B**) Summary data depict the time course of normalized N1 amplitude following 5 Hz CF stimulation (black arrow) in the control (open circles) and C21 (1 μM; filled circles) groups. The horizontal bar indicates the period of C21 application. (**C**,**D**) Mean values (±S.E.M) and individual data points show normalized N1 amplitude (**C**) and PPR (**D**) before and after tetanic stimulation in control and C21 groups. * *p* < 0.05 versus Pre; # *p* < 0.05 versus Post of control group. *n* = 8 recordings/8 slices/6 mice in the control group; *n* = 8 recordings/8 slices/6 mice in C21 group.

Consistent with previous studies [7,9], 5 Hz stimulation combined with C21 failed to induce detectable CF–PC LTD after mGluR1 was inhibited by JNJ in control mice (Figure 3A,B). At 30–40 min post-stimulation, the normalized N1 amplitude was 100.69 ± 2.97% of baseline (Pre: 100.0 ± 2.71%; F_(1,14)_ = 0.01, *p* = 0.91; Figure 3C). However, 5 Hz stimulation plus C21 rescued JNJ-abolished CF–PC LTD and raised the PPR in the hM3D (Gq) group (Figure 3A,B). At 30–40 min post-tetanic stimulation, normalized N1 amplitude declined to 76.96 ± 2.12% of baseline (Pre: 100.0 ± 2.58%; F_(1,14)_ = 47.60, *p* < 0.001; *n* = 8; Figure 3C) in the hM3D (Gq) group, which was significantly lower than values obtained in the control group (100.69 ± 2.97%; F_(1,14)_ = 42.24, *p* < 0.001; Figure 3C). The PPR was 0.75 ± 0.02 of the baseline level (Pre: 0.62 ± 0.02; F_(1,14)_ = 14.74, *p* = 0.002; *n* = 8 recordings; Figure 3D) in the hM3D (Gq) group, a value significantly greater than that measured in the control group (0.59 ± 0.02; F_(1,14)_ = 26.11, *p* < 0.001; Figure 3D). These results demonstrate that upon blockade of mGluR1-dependent LTD, chemogenetic activation of LC noradrenergic fibers enables tetanic stimulation to induce a novel form of CF–PC LTD in the mouse cerebellar cortex.

**Figure 4:**
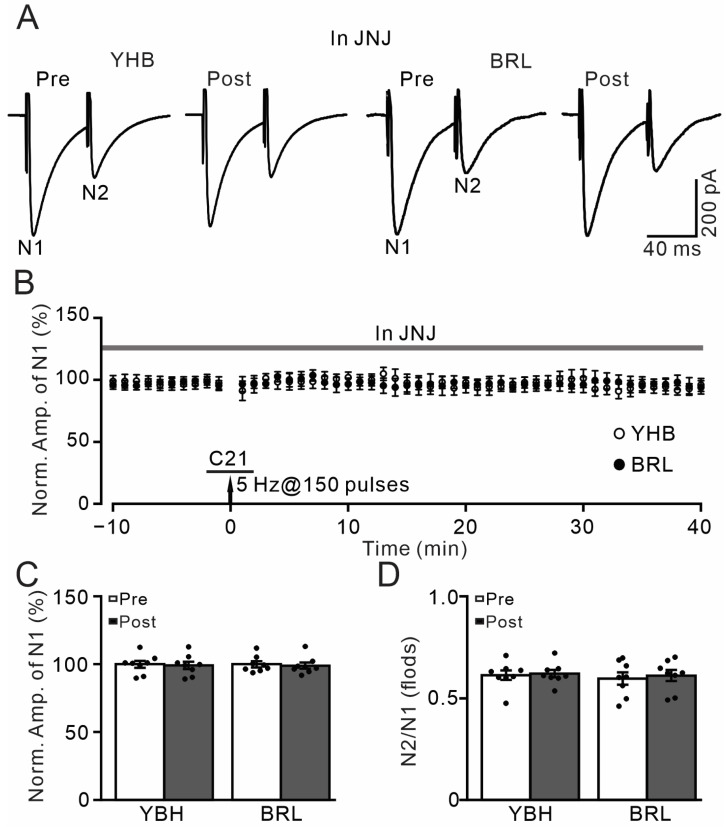
In the presence of JNJ (1 μM), chemogenetic activation of LC noradrenergic afferents triggers CF–PC LTD via α_2_A-AR in the mouse cerebellar cortex. (**A**) Representative traces display CF–PC EPSCs elicited by CF paired-pulse stimulation (0.05 Hz, 0.2 ms) before and after 5 Hz tetanic stimulation (150 pulses) with chemogenetic activation of LC noradrenergic afferents in JNJ + YHB (10 μM) and JNJ + BRL (10 μM) groups. (**B**) Summary data depict the time course of normalized N1 amplitude following 5 Hz CF tetanic stimulation (black arrow) in JNJ + YHB (open circles) and JNJ + BRL (filled circles) groups. The horizontal bar indicates the period of C21 application. (**C**,**D**) Mean values (±S.E.M) and individual data points show normalized N1 amplitude (**C**) and PPR (**D**) before and after tetanic stimulation in each group. *n* = 8 recordings/8 slices/4 mice in JNJ + YHB group; *n* = 8 recordings/8 slices/4 mice in JNJ + BRL group.

Since α_2_-ARs are involved in the regulation of CF–PC synaptic transmission [20], we then explored whether the novel form of CF–PC LTD triggered by chemogenetic activation of LC noradrenergic fibers under mGluR1 blockade depends on α_2-_-AR signaling. In the presence of JNJ and YHB (α_2-_AR antagonist, 10 μM), tetanic stimulation failed to elicit CF–PC LTD (Figure 4A,B). During 30–40 min after tetanic stimulation, the normalized N1 amplitude was 99.31 ± 2.73% of the baseline level (Pre: 100.0 ± 2.53%; F_(1,14)_ = 0.03, *p* = 0.86; *n* = 8 recordings; Figure 4C), and the PPR was 0.63 ± 0.02 of the baseline level (Pre: 0.62 ± 0.02; F_(1,14)_ = 0.35, *p* = 0.57; *n* = 8 recordings; Figure 4D). These data demonstrate that presynaptic α_2_-AR activation mediates CF–PC LTD induced by chemogenetic stimulation of LC noradrenergic afferents in the mouse cerebellar cortex.

Furthermore, we observed if CF–PC LTD was triggered by chemogenetic activation of LC noradrenergic fibers under mGluR1 blockade via α_2_A-AR signaling. When α_2_A-ARs were blocked by the selective antagonist BRL (10 μM) [29], chemogenetic activation of LC noradrenergic afferents with C21 failed to elicit tetanic stimulation-induced CF–PC LTD (Figure 4A,B). In the presence of JNJ and BRL, the normalized N1 amplitude during 30–40 min after tetanic stimulation was 99.02 ± 2.26% of the baseline level (Pre: 100.0 ± 2.13%; F_(1,14)_ = 0.10, *p* = 0.76; *n* = 8 recordings; Figure 4C), and the PPR was 0.62 ± 0.02 of the baseline level (Pre: 0.61 ± 0.03; F_(1,14)_ = 1.80, *p* = 0.22; *n* = 8 recordings; Figure 4D). These findings indicate that chemogenetic activation of LC noradrenergic afferents triggers tetanic stimulation-evoked CF–PC LTD via presynaptic α_2_A-AR signaling in the mouse cerebellar cortex.

**Figure 5:**
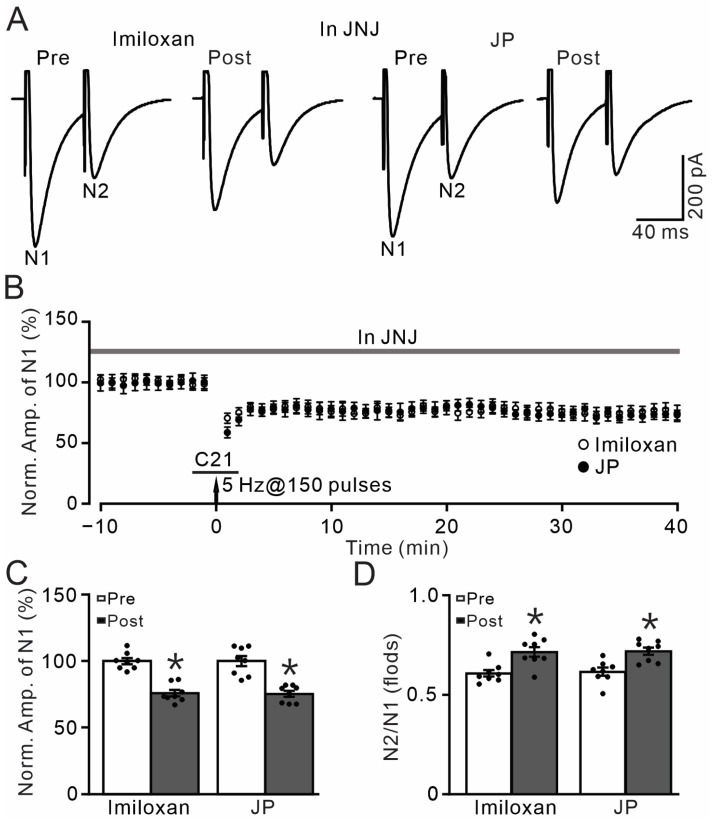
In the presence of JNJ, the CF–PC LTD triggered by chemogenetic activation of LC noradrenergic afferents is not mediated by α_2_B-AR or α_2_C-AR. (**A**) Representative traces show CF–PC EPSCs evoked by CF paired-pulse stimulation (0.05 Hz, 0.2 ms), recorded before and after 5 Hz stimulation (150 pulses) with C21 application in the JNJ + imiloxan (10 μM) and JNJ + JP (10 μM) groups. (**B**) Summary data demonstrate the time course of normalized N1 amplitude following 5 Hz CF tetanic stimulation (black arrow) in JNJ + imiloxan (open circles) and JNJ + JP (filled circles) groups. The horizontal bar indicates the period of C21 application. The horizontal bar indicates the period of C21 application. (**C**,**D**) Mean values (±S.E.M) and individual data points show normalized N1 amplitude (**C**) and PPR (**D**) before and after tetanic stimulation in each group. * *p* < 0.05 vs. Pre. *n* = 8 recordings/8 slices/6 mice in JNJ + imiloxan group; *n* = 8 recordings/8 slices/5 mice in JNJ + JP group.

Moreover, we examined whether CF–PC LTD triggered by chemogenetic activation of LC noradrenergic afferents depends on α_2_B-AR or α_2_C-AR signaling by bath-applying the α_2_B-AR antagonist imiloxan (10 μM) and the α_2_C-AR antagonist JP (10 μM) to cerebellar slices. Blockade of α_2_B-ARs or α_2_C-ARs failed to eliminate CF–PC LTD evoked by 5 Hz stimulation together with chemogenetic activation of LC noradrenergic afferents (Figure 5A,B). In the presence of JNJ and imiloxan, the normalized N1 amplitude at 30–40 min post-tetanic stimulation decreased to 75.78 ± 2.42% of baseline (Pre: 100.0 ± 2.25%; *n* = 8; F_(1,14)_ = 53.89, *p* < 0.001; Figure 5C), while the PPR increased to 0.72 ± 0.02 (Pre: 0.61 ± 0.02; *n* = 8; F_(1,14)_ = 25.38, *p* = 0.002; Figure 5D). In the presence of JNJ and JP co-applied, normalized N1 amplitude at 30–40 min post-tetanic stimulation declined to 75.39 ± 2.26% of baseline (Pre: 100.0 ± 3.77%; F_(1,14)_ = 31.37, *p* < 0.001; *n* = 8; Figure 5C), whereas PPR increased to 0.72 ± 0.02 (Pre: 0.62 ± 0.02; *n* = 8; F_(1,14)_ = 20.79, *p* = 0.003; Figure 5D). These findings show that blockade of neither α_2_B-ARs nor α_2_C-ARs abolishes CF–PC LTD triggered by chemogenetic activation of LC noradrenergic afferents, suggesting that C21-evoked CF–PC LTD is not mediated by α_2_B-ARs or α_2_C-ARs but relies predominantly on presynaptic α_2_A-AR signaling in the mouse cerebellar cortex.

### 3.3. Chemogenetic Activation of LC Noradrenergic Afferents Triggers Tetanic Stimulation-Evoked LTD of Glutamate Fluorescence via α_2_A-ARs

**Figure 6:**
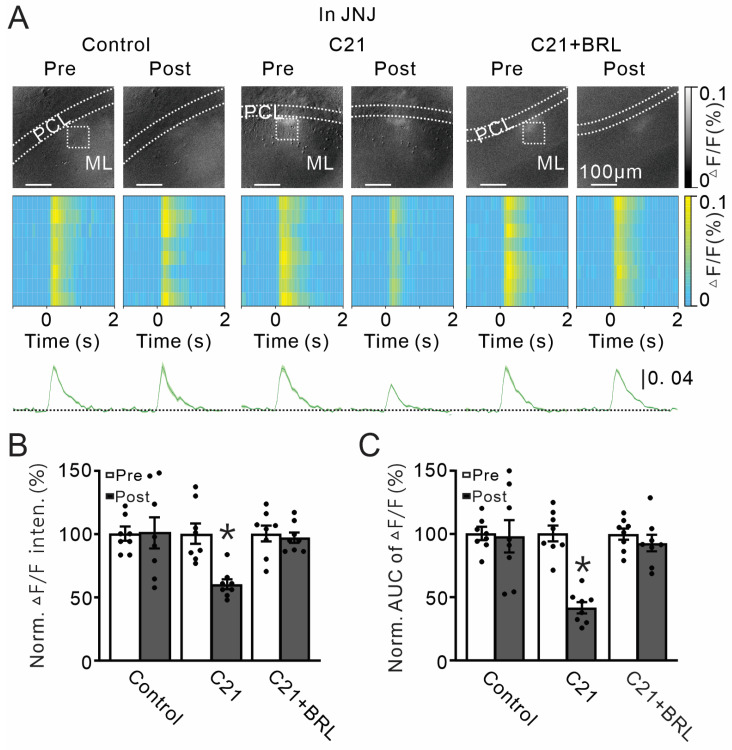
In the presence of JNJ (1 μM), chemogenetic activation of LC noradrenergic afferents triggers LTD of glutamate fluorescence via α_2_A-AR. (**A**). In the presence of JNJ (1 μM), representative images (**upper**), heatmaps (**middle**) and averaged traces (**lower**) show iGluSnFR fluorescence signals elicited by CF stimulation before (Pre) and after (Post) 5 Hz tetanic stimulation (150 pulses) in control, C21 and C21 + BRL (10 μM) groups. Yellow arrows mark the positions of the stimulating (Sti.) and recording (Rec.) electrodes. The boxes indicate the areas of the fluorescent response for analysis. The Purkinje cell layer (PCL) and molecular layer (ML) are separated by the white dotted line. (**B**,**C**) Mean values (±S.E.M) and individual data points show normalized ΔF/F intensity (**B**) and AUC (**C**) of glutamate fluorescence signals before and after the tetanic stimulation in each group. * *p* < 0.05 vs. Pre. *n* = 8 recordings/8 slices/5 mice in the control group; *n* = 8 recordings/8 slices/5 mice in C21 group; *n* = 8 recordings/8 slices/5 mice in C21 + BRL group.

To monitor glutamate dynamics at CF–PC synapses, we stereotaxically injected AAV2/9–hSyn–SF–iGluSnFR.A184S–WPRE into the cerebellar cortex to drive expression of the glutamate biosensor across the ML. We then recorded CF-evoked iGluSnFR.A184S fluorescence transients before and after tetanic stimulation during pharmacological blockade of mGluR1. In the presence of JNJ, 5 Hz tetanic stimulation failed to induce a sustained change in iGluSnFR.A184S fluorescence responses. However, the tetanic stimulation combined with C21 induced LTD of iGluSnFR.A184S fluorescence (Glu-LTD), characterized by a persistent reduction in the intensity and AUC of iGluSnFR.A184S fluorescence responses for more than 40 min (Figure 6A). At 30–40 min post-tetanic stimulation, normalized peak ΔF/F value in the C21-treated group declined to 60.2 ± 3.9% of baseline (Pre: 100.0 ± 8.0%; F_(1,14)_ = 15.18, *p* = 0.0059; *n* = 8; Figure 6B), a value markedly lower than that measured in the control group (100.6 ± 12.3%; F_(1,14)_ = 13.86, *p* = 0.0074; Figure 6B). Consistently, the normalized AUC of fluorescence traces fell to 41.5 ± 4.4% of baseline in the C21-treated cohort (Pre: 100.0 ± 6.1%; F_(1,14)_ = 71.7, *p* < 0.001; *n* = 8; Figure 6C), which was also significantly reduced relative to control values (97.7 ± 12.8%; F_(1,14)_ = 24.4, *p* = 0.0017; Figure 6C). These results demonstrate that following pharmacological inhibition of mGluR1, combined tetanic stimulation and chemogenetic activation of LC noradrenergic afferents induces presynaptic suppression of glutamate signaling at CF–PC synapses in the mouse cerebellar cortex.

Notably, in the presence of JNJ (1 μM) and BRL (10 μM), tetanic stimulation combined with C21 failed to trigger CF–PC Glu-LTD (Figure 6A,B). In the presence of JNJ and BRL, the normalized peak ΔF/F intensity during 30–40 min after tetanic stimulation was 96.7 ± 4.0% of the baseline level (Pre: 100.0 ± 6.3%; F_(1,14)_ = 0.3006, *p* = 0.6006; *n* = 8 recordings; Figure 6B), which was no significant difference from baseline. The normalized AUC of fluorescence responses was 92.9 ± 6.5% of the baseline (Pre: 100.0 ± 4.5%; F_(1,14)_ = 0.1.159, *p* = 0.3174; *n* = 8 recordings; Figure 6C), which was comparable to baseline. These results indicate that Glu-LTD at CF–PC synapses were induced by chemogenetic activation of LC noradrenergic afferents via α_2_A-AR in the mouse cerebellar cortex.

### 3.4. Chemogenetic Activation of LC Noradrenergic Afferents Triggers Tetanic Stimulation-Induced CF–PC LTD via the CDK5/PKA Signaling Cascade

**Figure 7:**
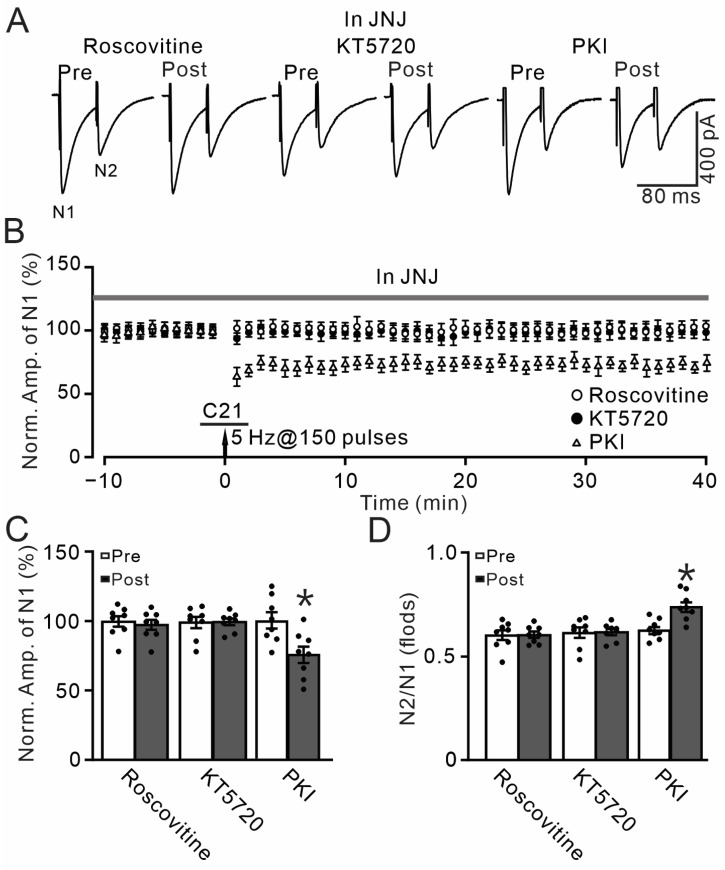
In the presence of JNJ, chemogenetic activation of LC noradrenergic afferents facilitates LTD of CF-triggered glutamate fluorescence through CDK5/PKA signaling pathway. (**A**) Representative traces show CF–PC EPSCs elicited by CF paired-pulse stimulation (0.05 Hz, 0.2 ms) before and after 5 Hz tetanic stimulation (150 pulses) with chemogenetic activation of LC noradrenergic afferents in JNJ + roscovitine (10 µM; CDK5 inhibitor), JNJ + KT5720 (10 nM; PKA inhibitor), and JNJ + PKI internal solution (PKI, 5μM) groups. (**B**) Summary data demonstrate the time course of normalized N1 amplitude following 5 Hz CF tetanic stimulation (black arrow) in JNJ + roscovitine (open circles), JNJ + KT5720 (filled circles) and JNJ + PKI internal solution (open triangles) groups. The horizontal bar indicates the period of C21 application. (**C**,**D**) Mean values (±S.E.M) and individual data points show normalized N1 amplitude (**C**) and PPR (**D**) before and after tetanic stimulation in each group. * *p* < 0.001 vs. Pre. *n* = 8 recordings/8 slices/5 mice in JNJ + roscovitine group; *n* = 8 recordings/8 slices/4 mice in KT5720 group, *n* = 8 recordings/8 slices/4 mice in PKI internal solution group.

Previous studies have demonstrated that CDK5 is tightly linked to neuronal synaptic plasticity, especially the regulation of presynaptic plasticity [30,31]. We thus tested whether CF–PC LTD triggered by chemogenetic activation of LC noradrenergic afferents is mediated by CDK5 signaling using the selective CDK5 inhibitor roscovitine [32]. To achieve effective CDK5 inhibition, roscovitine (20 μM) was added to ACSF 20 min before whole-cell recordings and present throughout the experiment. Co–perfusion with JNJ and roscovitine abolished CF–PC LTD induced by combined 5 Hz stimulation and C21 application (Figure 7A,B). At 30–40 min post-tetanic stimulation, the normalized N1 amplitude remained at 98.59 ± 3.42% of baseline (Pre: 100.0 ± 3.99%; F_(1,14)_ = 0.33, *p* = 0.58; *n* = 8; Figure 7C), showing no significant difference from baseline. The mean PPR was 0.61 ± 0.01, which was comparable to the pre-stimulation baseline (Pre: 0.61 ± 0.02; F_(1,14)_ = 0.01, *p* = 0.92; *n* = 8; Figure 7D).

We further investigated the involvement of PKA signaling in CF–PC LTD enhanced by chemogenetic activation of LC noradrenergic afferents using KT5720, a selective PKA inhibitor. KT5720 (10 nM) was bath-applied to ACSF 20 min before whole-cell recordings to achieve full PKA inhibition and remained present for the duration of experiments. When JNJ and KT5720 were co-perfused, 5 Hz tetanic stimulation combined with C21 treatment did not evoke measurable CF–PC LTD (Figure 7A,B). At 30–40 min post-tetanic stimulation, the normalized N1 amplitude stayed at 100.26 ± 3.48% of baseline (Pre: 100.0 ± 3.36%; F_(1,14)_ = 0.02, *p* = 0.90; *n* = 8; Figure 7C), with no significant change relative to baseline. The mean PPR (0.62 ± 0.01) was similar to the baseline level (Pre: 0.62 ± 0.02; F_(1,14)_ = 0.01, *p* = 0.92; *n* = 8; Figure 7D). These data indicate that CF–PC LTD induced by combined tetanic stimulation and chemogenetic activation of LC noradrenergic afferents is dependent on PKA signaling cascade in vitro in mice. Since the previous study demonstrated that CF–PC LTD depended on the postsynaptic PKA signaling pathway [33], we applied a membrane-impermeable PKA-inhibitory peptide, PKI (5 μM), in the intracellular solution of the recording pipette to determine whether CF–PC LTD triggered by chemogenetic activation of LC noradrenergic afferents relies on postsynaptic PKA activation. Recording from PCs with intracellular PKI solution failed to block CF–PC LTD induced by 5 Hz stimulation combined with JNJ and C21 treatment (Figure 7A,B). At 30–40 min after tetanic stimulation, the normalized N1 amplitude decreased to 76.28 ± 5.98% of baseline (Pre: 100.0 ± 5.88%; F_(1,14)_ = 43.70, *p* < 0.001; *n* = 8; Figure 7B), and the PPR was 0.74 ± 0.02, which was significantly higher than baseline (Pre: 0.63 ± 0.02; F_(1,14)_ = 20.27, *p* < 0.001; *n* = 8; Figure 7C). These data indicate that CF–PC LTD was induced by combined tetanic stimulation and chemogenetic activation of LC noradrenergic afferents via the presynaptic PKA signaling pathway in vitro in mice. Taken together, our data demonstrate that blocking CDK5 or PKA fully abolishes CF–PC LTD induced by combined tetanic stimulation and chemogenetic activation of LC-derived noradrenergic afferents, whereas selective blockade of postsynaptic PKA fails to abolish tetanic stimulation-induced CF–PC LTD, confirming that this form of synaptic plasticity relies on the presynaptic α_2_A-AR/CDK5/PKA signaling cascade in the mouse cerebellar cortex.

**Figure 8:**
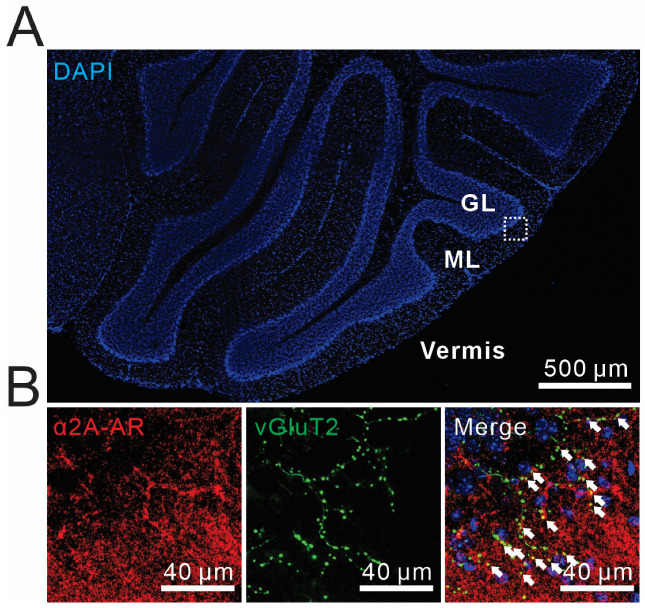
Co-localization of α_2_A-AR with vGluT2 at cerebellar CF terminals. (**A**) Representative confocal micrographs of mouse cerebellar sections stained with DAPI (blue, nuclear marker). (**B**) High-magnification views of the boxed area in panel A show α_2_A-AR immunolabeling and vGluT2-expressing CF terminals within the ML. Merged channels reveal co-localization between α_2_A-AR and vGluT2-positive CF terminals (white arrowheads). ML, molecular layer; GL, granule layer.

Finally, we examined the expression of α_2_A-ARs at CF terminals using immunohistochemistry combined with intraocular injection of AAV2/1–vGluT2–EGFP virus. Immunofluorescence signals for α_2_A-ARs and vGluT2 were detected with a high-speed multiphoton confocal laser-scanning microscope. As shown in Figure 8, α_2_A-AR immunolabeling was present throughout the cerebellar ML and colocalized with vGluT2-positive CF terminals (Figure 8B). These findings confirm α_2_A-AR expression at CF terminals and further demonstrate that chemogenetic activation of LC-derived noradrenergic afferents potentiates mGluR1-dependent CF–PC LTD via presynaptic α_2_A-AR-triggered Glu-LTD in mouse cerebellar slices in vitro.

## 4. Discussion

This study demonstrates that chemogenetic activation of LC noradrenergic afferents markedly potentiates 5 Hz-evoked CF–PC LTD in mouse cerebellar slices. Following mGluR1 blockade, activation of LC noradrenergic afferents elicits a novel form of CF–PC LTD, accompanied by an increased N2/N1 ratio. Importantly, both CF–PC synaptic LTD and CF-evoked glutamate fluorescence reduction were fully abolished by blockade of α_2_-AR or α_2_A-AR, yet remained unaffected following inhibition of α_2_B-AR or α_2_C-AR. Notably, pharmacological blockade of CDK5 or PKA entirely eliminated the LTD triggered by chemogenetic activation of LC noradrenergic afferents in mouse cerebellar slices. Immunofluorescence analyses showed strong α_2_A-AR expression on PC dendrites, and clear colocalization of α_2_A-AR with vGluT2 at CF terminals. These results indicate that activation of LC noradrenergic afferents potentiates CF–PC LTD by triggering Glu-LTD at CF terminals through the α_2_A-AR/CDK5/PKA signaling cascade in the mouse cerebellar cortex. These findings provide novel evidence that LC noradrenergic inputs regulate cerebellum-dependent functions, including motor learning and fine motor coordination, via CF–PC synaptic signaling.

### 4.1. Chemogenetic Activation of LC Noradrenergic Afferents Facilitates CF–PC LTD Through α_2_A-AR Signaling

The cerebellum acts not only as a central hub for processing signals transmitted by mossy fibers and CFs, but also as an integrative brain region regulated by widespread monoaminergic inputs such as NA. Noradrenergic afferents derived from the LC in the pons project broadly across the molecular, PC and granular layers of the cerebellar cortex. Accordingly, the firing of LC noradrenergic neurons modulates cerebellar circuit activity and synaptic function through distinct AR subtypes [14,17]. In the present study, chemogenetic activation of LC noradrenergic afferents markedly potentiated CF–PC LTD. This indicates that stimulation of hM3D(Gq)-expressing LC noradrenergic projections triggers robust endogenous NA release to modulate CF–PC synaptic plasticity in the cerebellar cortex. Since tetanus-evoked CF–PC LTD is mediated by mGluR1 signaling [9], we applied a selective mGluR1 antagonist to block mGluR1-dependent LTD. After mGluR1 inhibition, tetanic stimulation failed to induce CF–PC LTD. These results are consistent with prior reports [7,9], confirming that intact mGluR1 activity is essential for tetanus-evoked CF–PC LTD at baseline. Notably, chemogenetic activation of LC-derived noradrenergic afferents restored CF-GC LTD together with an increase in PPR, suggesting that LC noradrenergic inputs modulate CF–PC LTD in the mouse cerebellar cortex via ARs.

Modulation of CF–PC synaptic transmission by NA within the cerebellar cortex has been extensively characterized previously [20,21,22]. Under in vitro conditions, bath perfusion of NA attenuates CF-triggered dendritic Ca^2+^ transients in PCs via α_2-_-ARs, thereby regulating the induction of PF–PC associative synaptic plasticity [20]. Activation of α_2_-ARs reduces glutamate release from CF axon terminals, attenuates CF-evoked calcium transients in PC dendrites, and diminishes endocannabinoid release, thereby impairing long-term plasticity at PF–PC synapses [20]. Under in vivo conditions, topical NA application to the cerebellar surface depresses spontaneous complex spike firing in PCs and suppresses CF–PC synaptic transmission through the α_2_A-AR/PKA signaling cascade. The findings suggest that α_2_A-AR signaling tunes CF–PC synaptic strength by decreasing the probability of presynaptic glutamate release from CF terminals [21,22]. The present results demonstrate that the facilitation of CF–PC LTD induced by chemogenetic activation of LC noradrenergic afferents under mGluR1 blockade is abolished by pan-α_2_-AR antagonists, indicating that presynaptic α_2_-AR activation mediates this novel CF–PC LTD. Moreover, selective blockade of α_2_A-ARs eliminates the tetanus-evoked CF–PC LTD triggered by chemogenetic stimulation of LC noradrenergic afferents, indicating that LC noradrenergic afferent activation triggers tetanus-evoked CF–PC LTD via presynaptic α_2_A-AR signaling in the mouse cerebellar cortex. In addition, the CF–PC LTD triggered by chemogenetic LC noradrenergic afferents activation remained unaffected after blockade of α_2_B-AR or α_2_C-AR, confirming that the novel CF–PC LTD is independent of α_2_B/α_2_C-AR but predominantly relies on presynaptic α_2_A-AR signaling in the mouse cerebellar cortex. Importantly, immunofluorescence staining detected abundant α_2_A-AR immunoreactivity across the cerebellar ML, and α_2_A-AR signals exhibited strong colocalization with vGluT2-positive CF terminals. This anatomical evidence supports our functional results, demonstrating that presynaptic α_2_A-ARs at CF terminals mediate the facilitatory effect of LC noradrenergic afferent stimulation on the basal CF–PC LTD. Collectively, our findings indicate that activation of LC-derived noradrenergic afferents recruits a novel form of CF–PC LTD at CF terminals, thereby leading to enhanced overall depression of CF–PC synaptic transmission in mouse cerebellar slices.

### 4.2. Chemogenetic Activation of LC Noradrenergic Afferents Induces Tetanus-Evoked Glu-LTD at CF–PC Synapses via α_2_A-AR Signals

The genetically encoded glutamate fluorescent indicator iGluSnFR serves as a robust tool to directly monitor synaptic glutamate dynamics at excitatory synapses and quantify glutamate release originating from neurons and astrocytes [34]. Given that iGluSnFR reliably tracks fluctuations in extracellular glutamate concentration with high temporal resolution, this probe is extensively applied to quantify presynaptic glutamate release and its activity-dependent modulation [34,35]. Since CFs originate from the IO and form strong glutamatergic synapses onto PCs in the cerebellar cortex, we employed iGluSnFR.A184S to visualize CF-evoked glutamate transients and quantify long-term changes in presynaptic glutamate release from CF terminals after tetanic stimulation. Our results show that when mGluR1 was pharmacologically blocked, tetanic stimulation coupled with chemogenetic activation of LC noradrenergic afferents elicited Glu-LTD, which was accompanied by sustained decreases in both the intensity and AUC of iGluSnFR.A184S fluorescence signals. Given that iGluSnFR reliably reports activity-dependent fluctuations in extracellular glutamate levels [34,35], our observations indicate that chemogenetic activation of LC noradrenergic afferents suppresses glutamate release from CF terminals. Pharmacological blockade of α_2_A-ARs fully abolished the CF–PC Glu-LTD triggered by chemogenetic activation of LC noradrenergic neurons, indicating that sustained suppression of presynaptic glutamate release upon LC noradrenergic afferents stimulation is mediated via α_2_A-AR signaling. These results demonstrate that chemogenetic activation of LC-derived noradrenergic afferents diminishes presynaptic glutamate release from CF terminals via the α_2_A-AR signaling pathway, which subsequently promotes tetanus stimulation-evoked presynaptic Glu-LTD at CF–PC synapses and reveals that NA-α_2_A-AR signaling modulates basal excitatory transmission and sets the plasticity threshold by reducing glutamate release at CF terminals in the cerebellar cortex. Taken together, these results provide direct functional evidence that LC-derived noradrenergic signaling recruits an additional presynaptic form of glutamate-dependent LTD via α_2_A-ARs characterized by persistently reduced glutamate-release probability at CF terminals, which consequently increases the overall magnitude of CF–PC LTD in mouse cerebellar slices. Nevertheless, we cannot rule out the possibility that LC-derived noradrenergic signaling directly modulates postsynaptic CF–PC LTD within the mouse cerebellar cortex.

### 4.3. Activation of Noradrenergic Afferent Projections Triggers CF–PC LTD via the α_2_A–AR/CDK5/PKA Signaling Cascade

CDK5 is a serine/threonine kinase highly active in mature neurons and modulates neurotransmitter release, synaptic vesicle trafficking and synaptic plasticity [30,31]. Its recruitment to membrane-associated structures and synaptic compartments enables CDK5 to serve as a core signaling regulator of neuronal activity, protein phosphorylation and synaptic remodeling [31,36]. Aberrant CDK5 activity disrupts synaptic transmission and plasticity in mature neurons, and is implicated in learning and memory as well as the pathogenesis of various neurological disorders [36,37]. Importantly, CDK5/p35 signaling modulates cerebellar synaptic plasticity and motor coordination, pointing to a key role of CDK5 in regulating cerebellar synaptic function [38]. The present data show that blockade of CDK5 with roscovitine fully abolished CF–PC LTD triggered by chemogenetic activation of LC noradrenergic projections. Consistent with a previous report [38], our findings confirm that CDK5 signaling is essential for the LC noradrenergic activity-dependent induction of CF–PC LTD. CDK5 modulates presynaptic synaptic vesicle cycling, mobilization, exocytosis and endocytosis, as well as the activity of voltage-gated Ca^2+^ channels, which collectively shapes neurotransmitter release probability [30,36]. In addition, CDK5 phosphorylates synapsin I to regulate vesicle partitioning between the reserve and recycling pools, thereby altering vesicle mobilization and presynaptic release probability [39]. Consistent with the iGluSnFR recordings, our findings indicate that α_2_A-AR and CDK5 function along the same presynaptic regulatory axis to mediate a long-term reduction in glutamate release from CF terminals. Given the well-documented roles of CDK5 in regulating synaptic vesicle mobilization, recycling pool stability and release probability, these results suggest that activation of the LC–NA system triggers α_2_A-AR-dependent CDK5 signaling to modify vesicle release properties at CF terminals. This reduces glutamate release and promotes the induction of presynaptic CF–PC LTD.

α_2_-ARs are Gi/o protein-coupled metabotropic receptors. Their activation suppresses AC activity, downregulates cyclic adenosine monophosphate (cAMP)/PKA signaling, and inhibits voltage-gated Ca^2+^ channels and presynaptic neurotransmitter release [40,41,42]. Of all α_2_-AR subtypes, α_2_A-AR acts as the primary mediator of noradrenergic negative feedback and presynaptic regulation of neurotransmitter release. Activation of α_2_A-AR suppresses excitatory synaptic transmission in the prefrontal cortex via the Gi–cAMP–PKA signaling cascade, thereby modulating neuronal network activity [42,43]. In the cerebellar cortex, NA selectively attenuates CF-evoked dendritic Ca^2+^ signals in PCs through α_2_-ARs, altering the instructive function of CF inputs in PF–PC associative synaptic function [20]. Our previous in vivo results have shown that NA depresses spontaneous complex spike firing in mouse cerebellar PCs through α_2_-ARs [21,22]. In acute cerebellar slices, NA further acts on presynaptic α_2_-ARs to inhibit CF–PC synaptic transmission via the AC–PKA signaling pathway [21,22]. We here show that pharmacological blockade of PKA fully abolished CF–PC LTD triggered by chemogenetic activation of LC noradrenergic afferents, indicating that the induction of presynaptic CF–PC LTD driven by LC noradrenergic activity relies on the PKA signaling cascade. A previous study demonstrated that CF–PC LTD also depends on the postsynaptic PKA signaling cascade [33]. However, our present results show that LC-triggered Glu-LTD is abolished by bath application of a PKA inhibitor but not by intracellular inhibition of PKA, indicating that CF–PC LTD triggered by chemogenetic activation of LC-derived noradrenergic afferents relies on activation of a presynaptic signaling cascade.

CDK5 maintains hippocampal LTP by regulating the cAMP/PKA signaling pathway. Genetic deletion of CDK5 reduces cAMP levels and inhibits PKA activity, which consequently impairs synaptic plasticity and cognitive function. Accumulating evidence suggests that CDK5 participates in the induction of both LTP and LTD, allowing it to exert bidirectional regulation of synaptic plasticity depending on brain region, cell type and stimulation paradigm [44]. Taken together, our results show that pharmacological blockade of either CDK5 or PKA completely abolishes CF–PC LTD triggered by chemogenetic activation of LC noradrenergic afferents. These findings indicate that this form of LTD relies on the α_2_A–AR/CDK5/PKA signaling cascade in the mouse cerebellar cortex.

### 4.4. Physiological Significance, Limitations and Future Directions

In this study, 5 Hz facial stimulation-induced CF–PC LTD persistently reduces the amplitude of CF–PC synaptic responses without changing the paired-pulse ratio, confirming that this basal form of LTD is postsynaptically expressed [7,9,11]. However, chemogenetic activation of LC noradrenergic afferents triggers a novel LTD through the presynaptic α_2_A-AR/CDK5/PKA signaling cascade in mouse cerebellar slices in vitro. These findings indicate that presynaptic depression induced by LC-derived noradrenergic afferent activation is sufficiently mild to spare the induction of mGluR1-dependent postsynaptic LTD, enabling the superimposition of presynaptic LTD onto postsynaptic LTD. Collectively, these results demonstrate that the activation of LC-derived noradrenergic afferents elicits an additional LTD component that diminishes neurotransmitter release, modifies synaptic dynamic characteristics, reshapes extrasynaptic signaling in the mouse cerebellar cortex, and contributes to modulation of cerebellum-dependent motor behaviors [7,8,10,11,12]. Furthermore, LC neurons are robustly activated during arousal, attention and stress, leading to widespread noradrenergic release across brain regions, and dynamically modulates synaptic plasticity in a behavioral state-dependent manner [4]. In this context, the presynaptic Glu-LTD of CF–PC synapses identified in the present study may represent a previously unrecognized cellular mechanism by which stress-associated LC activation fine-tunes cerebellar synaptic plasticity and information processing [45,46]. Moreover, dysregulated noradrenergic signaling is closely implicated in a variety of neurological and neuropsychiatric disorders, including anxiety, depression, autism spectrum disorder, stress-related cognitive dysfunction, and Alzheimer’s disease [47,48]. On the other hand, functional crosstalk between noradrenergic and dopaminergic systems serves as a core mechanism for the coordination of long-term synaptic plasticity during salient behavioral experiences [5,6] and odor discrimination learning [49].

The present work primarily used whole-cell patch-clamp recordings and iGluSnFR imaging in acute cerebellar slices. Acute brain slices represent a reliable experimental model for investigating local synaptic transmission and intracellular signaling, as they allow precise control of drug delivery, stimulation protocols and recording conditions. Nevertheless, ex vivo slice preparations inevitably disrupt long-range projections and native neural network connectivity. Accordingly, they cannot fully recapitulate the dynamic interplay of activity within the IO–CF–PC pathway and cerebellar behavioral states in freely moving animals. Methodological studies have shown that although acute brain slices preserve local cellular architecture and partial synaptic microcircuits, they lack intact long-range afferent and efferent connections. Furthermore, synaptic transmission and the threshold for LTD induction can be modulated by multiple factors, including slice temperature, slicing-induced tissue injury, progressive decline in tissue viability, and the composition of ACSF [50,51]. Moreover, C21 application selectively activates LC noradrenergic terminals in cerebellar slices, rather than the entire LC–cerebellum projection circuit. Accordingly, our observations mainly reflect local effects of LC noradrenergic terminal activation on CF–PC synaptic transmission and glutamate release, and do not fully recapitulate the global modulation of the cerebellar CF–PC pathway driven by LC neuronal activity in vivo. Future studies combining fiber photometry, NA sensors, in vivo electrophysiology and two–photon imaging in awake animals will help clarify how LC activation regulates cerebellar NA dynamics, CF-evoked complex spikes, CF–PC glutamate release, motor learning and interactions with dopaminergic circuits under both physiological and pathological conditions.

## 5. Conclusions

The LC sends extensive noradrenergic innervation to the cerebellar cortex, where NA modulates neuronal circuit activity via distinct AR subtypes. Our results demonstrate that α_2_A-ARs are abundantly expressed in the ML of the mouse cerebellar cortex and show prominent colocalization with CF terminals. Furthermore, chemogenetic activation of LC-derived noradrenergic projections recruits a novel form of CF–PC LTD that is expressed in parallel with tetanic stimulation-evoked, mGluR1-dependent CF–PC LTD, thereby producing a greater overall depression of CF–PC synaptic transmission. In addition, glutamate sensor imaging revealed that chemogenetic stimulation of LC noradrenergic afferents triggered LTD of glutamate fluorescence after mGluR1-dependent CF–PC LTD was blocked. These findings indicate that activation of LC-derived noradrenergic projections facilitates mGluR1-dependent CF–PC LTD by inducing α_2_A-AR-dependent depression of glutamate release from CF terminals in the in vitro mouse cerebellar cortex.

Notably, pharmacological blockade of either CDK5 or presynaptic PKA, but not postsynaptic PKA, completely abolished the LC-triggered CF–PC LTD, indicating that the presynaptic α_2_A-AR/CDK5/PKA signaling pathway is required for noradrenergic modulation of CF–PC long-term plasticity. These findings reveal a previously uncharacterized mechanism whereby activation of LC-derived noradrenergic afferents recruits a distinct presynaptic LTD at CF terminals and provides new insights into neuromodulatory control of cerebellar cortical function.

## Data Availability

The datasets generated and analyzed during the current study are available from the corresponding author on reasonable request.
